# Immunomodulatory Effects of the Neuropeptide Pituitary Adenylate Cyclase-Activating Polypeptide in Acute *Toxoplasmosis*

**DOI:** 10.3389/fcimb.2019.00154

**Published:** 2019-05-28

**Authors:** Caio Andreeta Figueiredo, Henning Peter Düsedau, Johannes Steffen, Nishith Gupta, Miklos Pal Dunay, Gabor K. Toth, Dora Reglodi, Markus M. Heimesaat, Ildiko Rita Dunay

**Affiliations:** ^1^Medical Faculty, Institute of Inflammation and Neurodegeneration, Otto-von-Guericke University Magdeburg, Magdeburg, Germany; ^2^Faculty of Life Sciences, Institute of Biology, Humboldt University, Berlin, Germany; ^3^Department and Clinic of Surgery and Ophthalmology, University of Veterinary Medicine, Budapest, Hungary; ^4^Department of Medical Chemistry, University of Szeged, Szeged, Hungary; ^5^Department of Anatomy, MTA-PTE PACAP Research Team, University of Pecs Medical School, Pecs, Hungary; ^6^Department of Microbiology and Hygiene, Charité - University Medicine Berlin, Berlin, Germany; ^7^Center for Behavioral Brain Sciences - CBBS, Magdeburg, Germany

**Keywords:** pituitary adenylate cyclase-activating polypeptide (PACAP), *Toxoplasma gondii*, acute infection, monocytes, macrophages, innate immunity, neurotrophins

## Abstract

Pituitary Adenylate Cyclase-Activating Polypeptide (PACAP) is an endogenous neuropeptide with distinct functions including the regulation of inflammatory processes. PACAP is able to modify the immune response by directly regulating macrophages and monocytes inhibiting the production of inflammatory cytokines, chemokines and free radicals. Here, we analyzed the effect of exogenous PACAP on peripheral immune cell subsets upon acute infection with the parasite *Toxoplasma gondii (T. gondii)*. PACAP administration was followed by diminished innate immune cell recruitment to the peritoneal cavity of *T. gondii*-infected mice. PACAP did not directly interfere with parasite replication, instead, indirectly reduced parasite burden in mononuclear cell populations by enhancing their phagocytic capacity. Although proinflammatory cytokine levels were attenuated in the periphery upon PACAP treatment, interleukin (IL)-10 and Transforming growth factor beta (TGF-β) remained stable. While PACAP modulated VPAC1 and VPAC2 receptors in immune cells upon binding, it also increased their expression of brain-derived neurotrophic factor (BDNF). In addition, the expression of p75 neurotrophin receptor (p75^NTR^) on Ly6C^hi^ inflammatory monocytes was diminished upon PACAP administration. Our findings highlight the immunomodulatory effect of PACAP on peripheral immune cell subsets during acute *Toxoplasmosis*, providing new insights about host-pathogen interaction and the effects of neuropeptides during inflammation.

## Introduction

Pituitary adenylate cyclase-activating polypeptide (PACAP) is a 38-amino-acid neuropeptide in the glucagon superfamily together with secretin and vasoactive intestinal peptide (VIP) (Sherwood et al., 2000). PACAP is widely expressed in the peripheral and central nervous systems (CNS) and functions as a neurotransmitter, neuromodulator and neurotrophic factor (Waschek, 2002; Zhou et al., 2002; Dejda et al., 2005; Botia et al., 2007; Armstrong et al., 2008; Abad and Tan, 2018). The nervous and immune systems participate in a complex bidirectional cross-talk through neuropeptides such as PACAP/VIP in multiple organs (Abad and Tan, 2018). Correspondingly, PACAP strongly improves the outcome of inflammatory disorders, such as rheumatoid arthritis, septic shock, inflammatory bowel disease, and multiple sclerosis in rodent models (Abad et al., 2001, 2003; Martinez et al., 2002; Gonzalez-Rey et al., 2006; Tan et al., 2009).

PACAP acts by binding to three specific membrane receptors from the G protein-coupled receptors (GPCR) family: PAC1, VPAC1, and VPAC2. The PAC1 receptor is highly expressed in the nervous system and possesses the highest affinity for PACAP, while the receptors VPAC1 and VPAC2 have the same lower affinity for PACAP, and are expressed among different cell types (Pozo et al., 1997). The majority of immune cells express one or more PACAP receptors. For example, PAC1 is expressed on peritoneal macrophages, microglia and pulmonary dendritic cells (DCs) (Delgado et al., 2004a). VPAC1 is constitutively expressed in T cells, macrophages, monocytes and DCs (Delgado et al., 2004a). VPAC2 is rarely expressed in these cells during a resting state, but its expression is induced following lipopolysaccharide (LPS) stimulation *in vitro* (Delgado et al., 1996b). Recently, the expression of VPAC1 and VPAC2 was found in innate lymphoid cells (ILC) 2 (Nussbaum et al., 2013), and has been implicated in the resolution of inflammation (Talbot et al., 2015).

Many studies have demonstrated that PACAP acts as a neuronal growth factor during development and regeneration (Waschek, 2002; Deguil et al., 2007; Watanabe et al., 2007). The neuromodulatory properties of PACAP were shown to be involved with the neurotrophin signaling of brain-derived neurotrophic factor (BDNF), promoting neuronal survival and synaptic plasticity (Frechilla et al., 2001). The family of neurotrophins is comprised of four secreted proteins, characterized by their ability to modulate survival, differentiation, and apoptosis of neurons (Bothwell, 2016). BDNF, nerve growth factor (NGF), neurotrophin-3 (NT-3), and neurotrophin-4 (NT-4) exert their functions via interaction with tropomyosin receptor kinases (Trk) TrkA, TrkB, TrkC, and the p75 neurotrophin receptor (p75^NTR^). The effect of PACAP on neurotrophin signaling was presented in human monocytes, where the exposure to the neuropeptide resulted in pro-inflammatory cell activation with increased Ca^2+^ mobilization (El Zein et al., 2006, 2007, 2008, 2010).

As an immunomodulator, PACAP exerts a dual role in regulating innate immunity depending on the activation status of cells and their environment. Several studies reported that PACAP is a potent immunomediator for both innate and adaptive immunity, primarily assuming an anti-inflammatory role. Exposure to PACAP inhibits the pro-inflammatory response of macrophages, such as the production of tumor necrosis factor (TNF) (Delgado et al., 1999b) and interleukin 6 (IL-6) (Martinez et al., 1998a), as well as the chemokines MCP-1 (CCL2), MIP1-α (CCL3) and RANTES (Delgado et al., 2003). Additionally, PACAP treatment leads to polarization of T helper cells to a Type 2 (Th2) phenotype (Delgado, 2003). It also promotes the development of tolerogenic DCs and favors the generation of regulatory T cells (Tregs), suppressors of immune responses (Delgado et al., 2005). In contrast, PACAP was able to stimulate the phagocytic activity, adhesion and mobility of resting macrophages as well as release of free radicals and IL-6 (Delgado et al., 1996a; Garrido et al., 1996; Martinez et al., 1998b), associating PACAP with a crucial mechanism to pathogen elimination.

Antiparasitic effects of PACAP were first described against the protozoa *Trypanosoma brucei*, showing a membrane-lytic effect, closely associated with autophagy and apoptosis-like cell death (Delgado et al., 2009). More recently, we showed that administration of PACAP ameliorated acute small intestinal inflammation and extra-intestinal sequelae caused by *Toxoplasma gondii* (*T. gondii*) infection (Heimesaat et al., 2014; Bereswill et al., 2019). *Toxoplasma gondii* is an obligate intracellular parasite acquired by oral ingestion of contaminated food or water. The parasite infects the small intestine and then differentiates to its rapidly replicating stage (tachyzoite), which is able to infect all nucleated cells through active penetration (Dobrowolski and Sibley, 1996). After crossing the intestinal barrier, the parasites encounter both resident and recruited immune cells, resulting in parasite elimination, antigen presentation and cytokine production (Buzoni-Gatel et al., 2006).

The successful dissemination of *T. gondii* within the host is highly dependent on invading migratory immune cells and the ability of these immune cells to phagocyte the parasite. Elimination of *T. gondii* involves a complex recruitment of immunity-related GTPases (IRGs) and guanylate-binding proteins (GBPs) (Zhao et al., 2009; Fentress et al., 2010). After infection, these host defense factors are known to accumulate in the membrane of the parasitophorous vacuole (PV) culminating with its disruption and parasite elimination (Macmicking, 2012). Moreover, a particular preference for myeloid cells has been explored as a key mechanism for parasite dissemination into the CNS (Weidner and Barragan, 2014; Blanchard et al., 2015).

Our group has previously demonstrated the critical importance of myeloid cells controlling *T. gondii* infection in the periphery as well as in the CNS (Dunay et al., 2008, 2010; Biswas et al., 2015; Mohle et al., 2016). Besides, we recently showed the innate immune response and the influence of neurotrophin signaling upon *T. gondii*-induced neuroinflammation. Particularly, neurotrophin signaling via p75^NTR^ altered innate immune cell behavior and changed the structural plasticity of neurons (Dusedau et al., 2019).

Here, we set out to evaluate the immunomodulatory effects of PACAP on the innate immune response during *T. gondii* acute infection. We show that PACAP is able to reduce immune cell recruitment and enhance phagocytic capacity of mononuclear cells, promoting parasite elimination. At the same time, the neuropeptide attenuated pro-inflammatory mediators while upregulating its own receptors. Interestingly, we detected altered expression of BDNF and p75^NTR^ in peritoneal cells, pointing toward the contribution of PACAP to the parasite elimination and neurotrophin signaling in immune cells upon acute *Toxoplasmosis*.

## Materials and Methods

### Animals

Experiments were conducted with female C57BL/6JRj mice (8 weeks old, purchased from Janvier, Cedex, France). All animals were group-housed in a 12 h day/night cycle at 22°C with free access to food and water under specific-pathogen-free conditions, according to institutional guidelines approved by the Animal Studies Committee of Saxony-Anhalt.

### *T. gondii in vitro* Culture

Tachyzoites of ME49 and PTG-GFP type II strain of *T. gondii* were grown in monolayers of human foreskin fibroblast (HFF) cells cultured in DMEM medium (FG0435, Biochrom, Germany), supplemented with 10% fetal bovine serum (FBS) (Thermo Fisher, Germany), 1% Penicillin/Streptomycin (Pen/Strep; Sigma, USA) and 1% non-essential amino acids (NEEA) (Thermo Fisher, Germany) (Morisaki et al., 1995). HFF cells and tachyzoites were scrapped from culture flasks, spinned down at 500 × g for 10 min and passed through 20 and 22G needles to liberate intracellular parasites. To obtain a host cell-free parasite suspension, the solution was filtered through a 5 μm Millex-SV syringe filter (Millipore, Germany). The parasite suspension was pelleted at 800 × g for 20 min, resuspended in 1 ml sterile phosphate-buffered saline (PBS) and the number of living tachyzoites was determined by counting under a light microscope using Trypan Blue 0.4%. Subsequently, the freshly egressed parasite suspension was used for infections and plaque assay experiments.

### Experimental Acute *T. gondii* Infection and PACAP Administration

In order to investigate acute *T. gondii* infection in the peritoneal cavity, all mice were infected by intraperitoneal (i.p.) injection of 1 × 10^4^ ME49 or PTG-GFP tachyzoites, freshly harvested from HFF cultures, in a final volume of 200 μl with PBS. For PACAP treatment, 50 μg (11 nmol/mouse) of the synthesized neuropeptide PACAP38 dissolved in 200 μl of PBS was administrated i.p. on days 2 and 4 post infection. Non-treated control mice received PBS only. At day 5 post infection (dpi) peritoneal exudate cells were collected by peritoneal lavage (Fentress and Sibley, 2011) for further analysis. Spleens were collected and stored in Allprotect Tissue Reagent (Qiagen, Germany) at −80°C until further processing.

### Flow Cytometric Analysis

Single cell suspensions were first incubated with ZOMBIE NIR™ fixable dye (BioLegend, San Diego, CA) or 7-AAD Viability Staining Solution (BioLegend) for live/dead discrimination. To prevent unspecific binding of antibodies, anti-FcγIII/II receptor antibody (clone 93) was applied to cells before staining with fluorochrome-conjugated antibodies against cell surface markers in FACS buffer (PBS, supplemented with 2% FBS and 0.1% sodium azide). CD11b (M1/70), Ly6C (HK1.4), MHCII I-A/I-E (M5/114.15.2), and F4/80 (BM8) were all purchased from eBioscience (San Diego, USA). Ly6G (1A8) and CD11c (N418) were purchased from BioLegend and p75^NTR^ (MLR2) from Abcam (Germany). Cells were incubated for 30 min at 4°C, washed and subsequently analyzed. Fluorescence Minus One (FMO) controls were used to determine the level of autofluorescent signals for each conjugated antibody. Data was acquired using a BD FACS Canto II (BD Biosciences, USA) or Attune NxT flow cytometer (Thermo Fisher, Germany) and analyzed using FlowJo (v10, FlowJo Inc., USA). A minimum of 2 × 10^5^ cells per samples were acquired.

### Plaque Assay

All experiments were conducted using fresh syringe-released extracellular tachyzoites. As previously described (Arroyo-Olarte et al., 2015), 200 parasites per well were used to infect HFF monolayers in six-well plates at different concentrations of PACAP. Briefly, parasitized cells were incubated for 7 days, fixed with cold methanol, and then stained with crystal violet. Plaques were imaged and scored for their sizes and numbers using the ImageJ software (NIH, US).

### Generation of Bone Marrow-Derived Macrophages

For generation of bone marrow-derived macrophages (BMDMs), femurs and tibias of 8 to 12 weeks old C57BL/6JRj mice were collected. Bones were flushed with a syringe filled with DMEM (FG0435, Biochrom, Germany) containing 10% FBS and 1% Pen/Strep to extrude bone marrow onto a 40 μm cell strainer. The obtained cell suspension was then spinned down for 10 min at 400 × g, 4°C and the cells were seeded into 6 well/plate using DMEM supplemented with 10% FBS, 1% Pen/Strep, 10 ng/ml recombinant murine GM-CSF (315-03, PeproTech, USA) and incubated at 37°C, 5% CO_2_. After 10 days, cells were primed to M1 macrophage phenotype with 150 Units/ml recombinant murine IFN-γ (315-05, Peprotech, USA) for 10 h, and then with 20 ng/ml of LPS (L2630, Sigma-Aldrich, USA) for another 12 h. Following stimulation, cells were washed and subsequently used for phagocytosis assay.

### *In vitro* Phagocytosis Assay

Phagocytosis assay was performed with M1 macrophages generated as described above and assessed in triplicates by incubation of cells with carboxylated, yellow-green fluorescent FluoSpheres™ (F8823, Fisher Scientific, Germany) in serum-free DMEM (with 1% Pen/Strep) in the presence of PACAP (0.1, 1, and 10 μM) for 4 h at 37°C, 5% CO_2_. Negative controls were established by keeping cells at 4°C throughout the experiment. After incubation, cells were washed twice with PBS to remove remaining microspheres before detachment with Accutase® solution (423201, BioLegend). Finally, detached cells were washed with PBS and directly stained with fluorochrome-conjugated antibodies for flow cytometric analysis. *In vitro* phagocytosis of microspheres was assessed by the median fluorescence intensity (MFI) of the positive population in the FITC-fluorescence channel. Further, percentages of cells in the FITC-positive fraction where divided according to the amount of microspheres internalized.

### DNA and RNA Isolation

DNA and RNA samples were isolated from peritoneal exudate cells and spleens of acutely infected mice. Spleen samples were homogenized in lysis buffer using BashingBeads Lysis tubes (Zymo Research, Germany) and isolated using AllPrep DNA/RNA Mini Kit (Qiagen, Germany) according to the manufacturer's instructions. For peritoneal exudate cells, parts of the cell suspensions were pelleted down, resuspended in lysis buffer and processed as describe above. The concentration and purity of DNA and RNA samples was determined using NanoDrop 2000 spectrophotometer (Thermo Fisher; Germany).

### qPCR

Parasite burden was assessed in triplicates using 30 ng of isolated DNA, FastStart Essential DNA Green Master and LightCycler^®^ 96 System (both Roche, Germany), as described previously (Biswas et al., 2017). Thermal-cycling parameters were set as follows: initial activation (95°C, 10 min), 45 amplification cycles consisting of denaturation (95°C, 15 s), annealing (60°C, 15 s) and elongation (72°C, 15 s). The DNA target was the published sequence of the highly conserved 35-fold-repetitive B1 gene of *T. gondii* (Burg et al., 1989; Lin et al., 2000). Murine argininosuccinate lyase (*Asl*) was used as reference gene for normalization and relative DNA levels were determined by the ratio *gene of interest / reference gene* and subsequently normalized to mean values of control group (Butcher et al., 2011). Primers were synthetized by Tib MolBiol (Germany) and used at 300 nM final concentration.

### RT-qPCR

Expression levels of cytokines, inflammatory mediators, host-defense factors, neurotrophins, and neurotrophin receptors were assessed in triplicates using 30 ng isolated RNA, TaqMan^®^ RNA-to-C_T_™ *1-Step* Kit (Applied Biosystems, Germany) and LightCycler^®^ 96 (Roche, Germany) as previously described (Mohle et al., 2016). Thermal-cycling parameters were set as follows: reverse transcription (48°C, 15 min), inactivation (95°C, 10 min) followed by 45 cycles of denaturation (95°C, 15 s) and annealing/extension (60°C, 1 min). Utilized TaqMan^®^ Gene Expression Assays (Applied Biosystems, Germany) are listed in Supplementary Table 1. *Hprt* was chosen as a reference gene and relative mRNA levels were determined by the ratio *gene of interest/reference gene* and subsequently normalized to mean values of control group.

The expression of PACAP receptors were evaluated using *Power* SYBR^®^ Green RNA-to-CT™ 1-Step Kit (Applied Biosystems, Germany). Samples were analyzed in triplicates (30 ng of isolated mRNA per reaction) using LightCycler^®^ 96 and the following parameters: reverse transcription (48°C, 30 min), inactivation (95°C, 10 min) followed by 55 cycles of denaturation (95°C, 15 s) and annealing/extension (60°C, 1 min) and melting curve analysis. The primer sequences are listed in Supplementary Table 1 and were synthetized by Tib MolBiol and used at 100 nM final concentration. Expression of *Hprt* was chosen as reference gene and relative mRNA levels were determined by the ratio *gene of interest / reference gene* and subsequently normalized to mean values of control group.

### Statistical Analysis

Results were statistically analyzed using GraphPad Prism 7 (STATCON, Germany) and two-tailed unpaired *t*-test was used on flow cytometry, qPCR and RT-qPCR data, and considered significant for *p* ≤ 0.05. Statistical analysis of phagocytosis assay data was carried out by applying one-way ANOVA with *post-hoc* Holm-Sidak test. For plaque assay, data was analyzed by one-way ANOVA followed by *post-hoc* Bonferroni test. All data are presented as arithmetic mean ± standard error of the mean (SEM) and are representative of two to three independent experiments.

## Results

### Immune Cell Recruitment Is Reduced Following PACAP Administration

**Figure 1:**
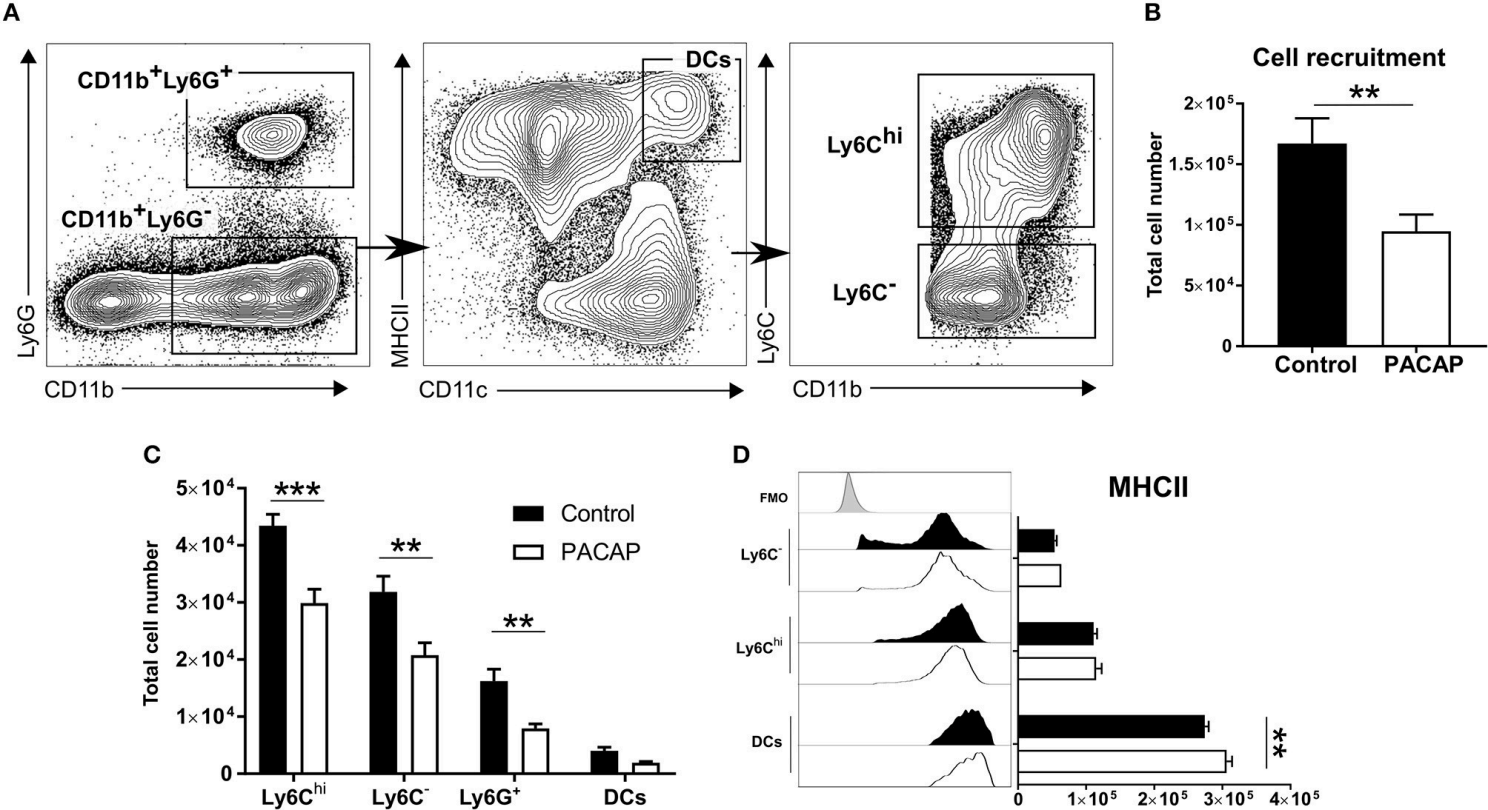
Immune cell recruitment and activation upon PACAP administration. Peritoneal cells of acutely-infected mice were analyzed by flow cytometry. Cells were selected based on the forward-scatter/side-scatter plot (FSS/SSC) and living, single cells were chosen for further analysis (not shown). **(A)** Gating strategy used to discriminate CD11b^+^Ly6G^+^ neutrophils and CD11b^+^Ly6G^−^ monocyte-derived cells. CD11b^+^Ly6G^−^ monocyte-derived cells were further divided into CD11c^hi^MHCII^hi^ DCs and then differentiated according to Ly6C expression: Ly6C^hi^ inflammatory monocytes and Ly6C^−^ peritoneal macrophages. **(B)** Shows the total recruitment of living single cells. **(C)** Bar charts show cell recruitment of the identified cell populations in peritoneal cavity exudate of control and PACAP-treated groups (control: black bars; PACAP-treated: white bars). **(D)** Histograms and bar charts show expression of the activation marker MHCII on peritoneal cell subsets according to median fluorescence intensity (MFI). Control (black bars/histogram) and PACAP-treated (white bars/histogram); FMO (gray histogram) data are expressed as mean ± SEM, **p < 0.01, ***p < 0.001 (two-tailed unpaired *t*-test).

Upon acute infection with *T. gondii*, neutrophil granulocytes, inflammatory monocytes and DCs are recruited to the site of infection (Robben et al., 2005; Dunay et al., 2008; Dunay and Sibley, 2010). To assess the effect of PACAP on cell recruitment and activation upon acute infection, mice were infected with tachyzoites, followed by administration of PACAP or PBS (control). The peritoneal exudate cells were collected and characterized by flow cytometry (Figure 1A). As our previous studies demonstrated the critical importance of myeloid cells in the control of *T. gondii* infection (Dunay et al., 2010; Biswas et al., 2015; Mohle et al., 2016), we analyzed the mononuclear compartment based on the expression of CD11b and Ly6G. While the CD11b^+^Ly6G^+^ subset defined neutrophil granulocytes, the fraction of CD11b^+^Ly6G^−^ cells was further discriminated into CD11c^hi^MHCII^hi^ DCs as previously described (Dupont et al., 2014). Subsequently, remaining immune cells were then defined according to Ly6C expression as Ly6C^hi^ inflammatory monocytes and Ly6C^−^ peritoneal macrophages.

In general, the PACAP-treated group presented less recruited cells in the peritoneal cavity (control: 1.67 × 10^5^ ± 0.10 × 10^5^ vs. PACAP: 0.95 × 10^5^ ± 0.07 × 10^5^; *p* = 0.0012) (Figure 1B). When compared to the controls, administration of PACAP significantly reduced the recruitment of all analyzed myeloid cell subsets. Ly6C^hi^ inflammatory monocytes appeared to be the most affected cell population (control: 4.34 × 10^4^ ± 0.10 × 10^3^ vs. PACAP: 2.99 × 10^4^ ± 2.41 × 10^3^; *p* = 0.00003), followed by Ly6C^−^ (control: 3.19 × 10^4^ ± 2.75 × 10^3^ vs. PACAP: 2.08 × 10^4^ ± 2.15 × 10^3^; *p* = 0.0003) and neutrophils (control: 1.62 × 10^4^ ± 2.09 × 10^3^ vs. PACAP: 7.96 × 10^3^ ± 7.7 × 10^2^; *p* = 0.0043). No evident difference was found for DCs (control: 4.02 × 10^3^ ± 6.10 × 10^2^ vs. PACAP: 1.92 × 10^3^ ± 2.29 × 10^2^; *p* = 0.4301) (Figure 1C). As the ability to present antigens is dependent on MHCII expression, we evaluated whether this was modulated by PACAP treatment upon acute *T. gondii* infection. Our data indicated that PACAP was able to increase the expression of MHCII on peritoneal DCs (control: 2.74 × 10^5^ ± 5.58 × 10^3^ vs. PACAP: 3.06 × 10^5^ ± 8.25 × 10^3^; *p* = 0.0012) (Figure 1D) but not on the other myeloid subsets (Ly6C^−^, control: 5.36 × 10^4^ ± 3.23 × 10^3^ vs. PACAP: 6.35 × 10^4^ ± 1.92 × 10^3^; *p* = 0.2448; Ly6C^hi^, control: 1.11 × 10^5^ ± 4.91 × 10^3^ vs. PACAP: 1.15 × 10^5^ ± 8.21 × 10^3^; *p* = 0.6767). Thus, PACAP reduced the recruitment of mononuclear cells to the peritoneal cavity upon *T. gondii* infection and increased MHCII expression on peritoneal DCs.

### Antiparasitic Effect of PACAP Is Immune Cell-Mediated

**Figure 2:**
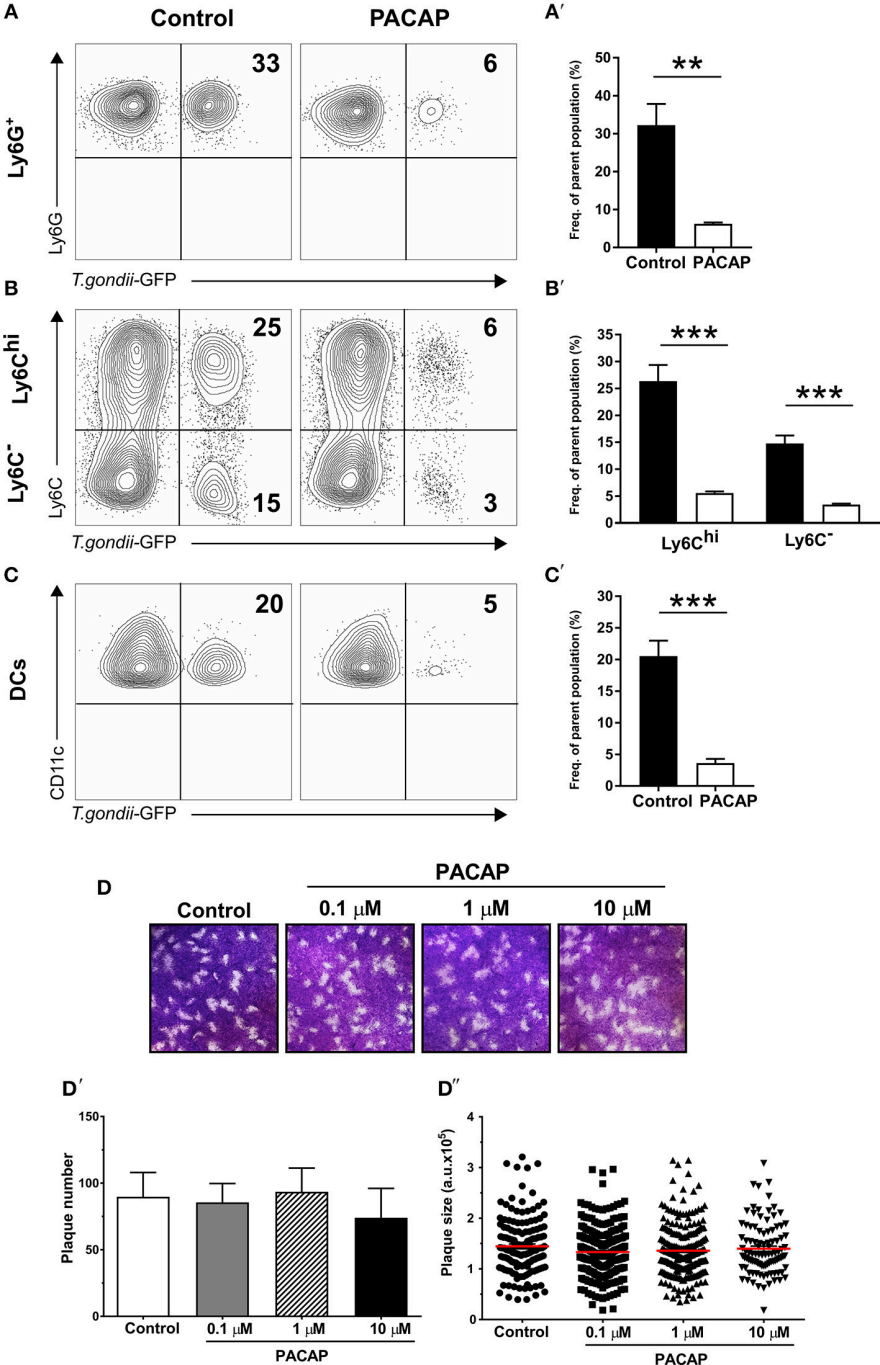
Anti-parasitic effect of myeloid peritoneal immune cells upon PACAP-treatment. Peritoneal cells of acutely-infected mice were isolated and analyzed by flow cytometry. A GFP-fluorescent *T. gondii* reporter was used to track the presence of the parasite in myeloid peritoneal subsets selected as described above. **(A–C)** Contour plots show the presence of GFP^+^ cells for each cell subset in control (left column) and PACAP-treated (right column) group. Numbers represent the mean percentage of parent population for each group from a representative experiment (*n* = 4). (**A′–C′**) Bar charts compare the frequency of GFP^+^ cells. Control (black bars) and PACAP-treated (white bars). **(D–D″)** Representative images show *in vitro* replication of *T. gondii* by plaque assays in the presence of PACAP. Plaques from three independent experiments were scored for size and numbers using ImageJ. **(D)** Images of the formed plaques initially infected with 200 tachyzoites/well; numbers indicate different PACAP concentration. **(D′)** Scored number of plaques under different concentrations of PACAP. **(D″)** Scored plaque sizes shown as arbitrary units (a.u.) in the presence of different PACAP concentrations. Data are expressed as mean ± SEM, **p < 0.01, ***p < 0.001 (two-tailed unpaired *t*-test).

Infected migratory immune cells, such as DCs, monocytes and macrophages are responsible for the parasite dissemination throughout host tissues, including lung, spleen, and also the peritoneal cavity (Ueno et al., 2014). As previous reports demonstrated the anti-parasitic effect of PACAP, we set out to analyze whether PACAP administration is able to affect the presence of *T. gondii* in myeloid-derived cell subsets. By infecting mice with a GFP-fluorescent reporter parasite, we were able to elucidate *T. gondii* occurrence in cell subsets isolated from the peritoneal cavity via flow cytometry. Here, the experimental group receiving PACAP showed a marked reduction of infected cells in all myeloid populations (Figures 2A′–C′). Alongside with Ly6G^+^ neutrophil granulocytes, Ly6C^hi^ monocytes represented the cell subset with the strongest reduction of parasitic GFP signal, while DCs and Ly6C^−^ macrophages were less affected (neutrophils, control: 32 ± 5.6% vs. PACAP: 6.2 ± 0.34%; *p* = 0.027; Ly6C^hi^, control: 26 ± 3% vs. PACAP: 5.5 ± 0.3%; *p* = 0.0004; Ly6C^−^, control: 15 ± 1.5% vs. PACAP: 3.4 ± 0.19%; *p* = 0.0003; DCs, control: 21 ± 2.5% vs. PACAP: 3.6 ± 0.65%; *p* = 0.0006) (Figures 2A–C). In order to address if this was mediated by the anti-parasitic effect of PACAP, we first performed an *in vitro* plaque assay using HFF culture infected with *T. gondii* in the presence of different PACAP concentrations (0.1, 1, and 10 μM) (Figure 2D). In contrast to our *in vivo* data in the peritoneal cavity, these results revealed that PACAP did not directly affect the size (Figure 2D′) or the number of plaques (Figure 2D″). Thus, PACAP has no direct effect on parasite elimination and/or impairment of the parasite development.

**Figure 3:**
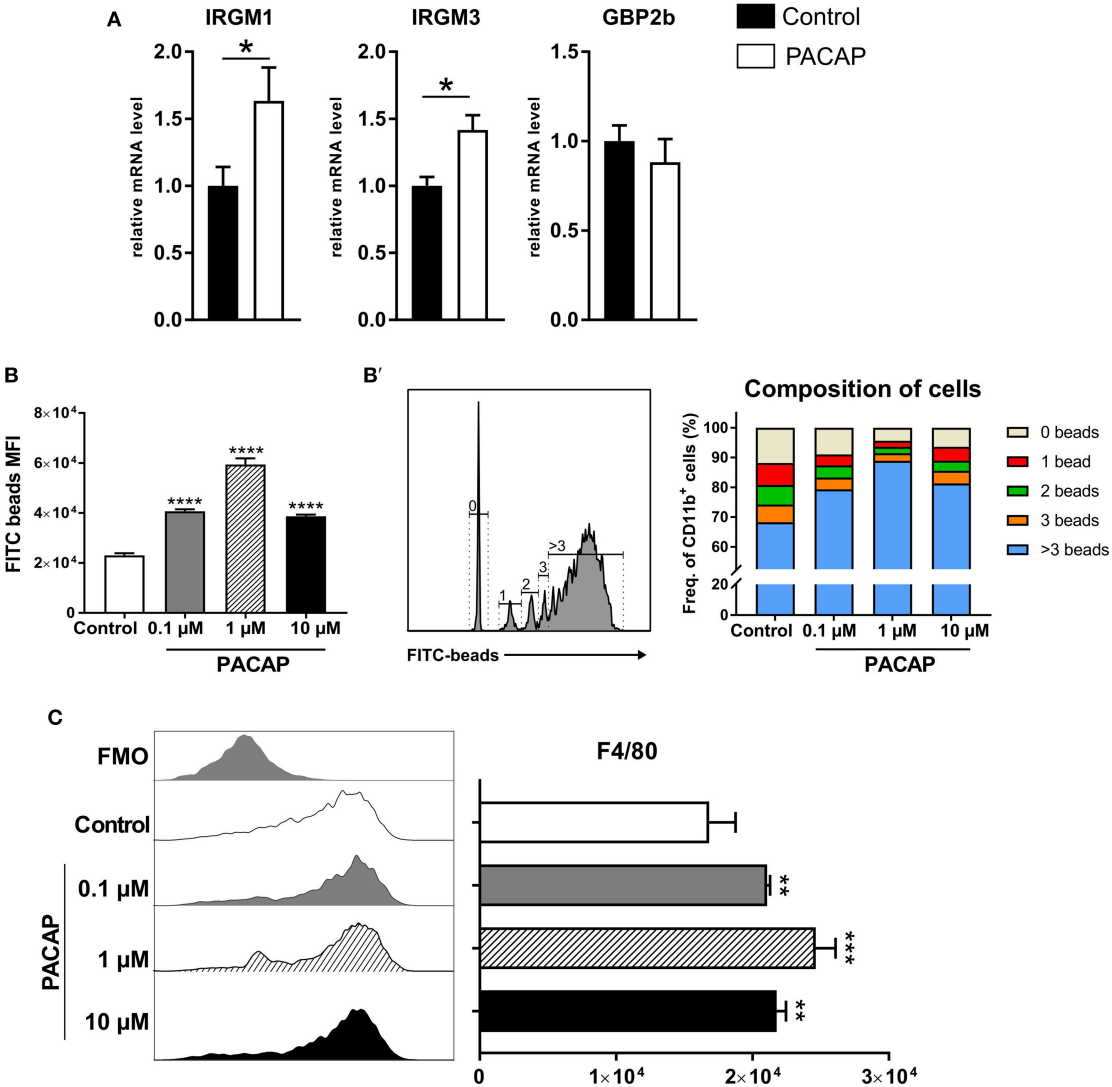
PACAP treatment increases expression of host-defense factors and phagocytosis. **(A)** Bar charts show gene expression of IRGs and GBP2b associated with parasite elimination in peritoneal exudate cells assessed by RT-qPCR. The gene expression levels of host defense factors were determined by RT-qPCR using *Hprt* as reference gene. Data were further normalized to mean values of control group; Control (black bars) and PACAP-treated (white bars). Bar charts present results as mean ± SEM, *n* = 4; *p < 0.05 (two-tailed unpaired *t*-test). **(B)** Phagocytosis of BMDMs primed to M1 phenotype was assessed by incubation with FITC-fluorescent microspheres in the presence of different PACAP concentrations (0.1, 1, and 10 μM); phagocytic capability was evaluated by the MFI of the positive populations. **(B')** Histograms and bar charts show frequency cells fractioned according to the amount of microspheres internalized. **(C)** Histograms and bar charts show activation marker F4/80 expressed on BMDMS in the phagocytosis assay. Data are expressed as mean ± SEM (one-way ANOVA with *post-hoc* Holm-Sidak test).

As infections with intracellular pathogens promote the expression of host defense factors such as IRGs and GBPs in myeloid cells, we hypothesized that these pathways were modulated by PACAP possibly explaining the reduced parasite burden *in vivo*. To this end, expression of IRGs (IRGM1, IRGM3) and GBP2b on peritoneal cells isolated from acutely infected mice was assessed by RT-qPCR (Figure 3A). Both IRGs were upregulated in the group that received PACAP (IRGM1: *p* = 0.0329; IRGM3: *p* = 0.0220), suggesting that the observed decrease in parasite burden might be associated with an improved phagocytic ability, also reported to be modulated by PACAP (Delgado et al., 1996a). Therefore, we performed, a phagocytosis assay with bone marrow-derived macrophages (BMDMs) primed to a classically activated M1 phenotype and stimulated with different concentrations of PACAP (Figure 3B). When compared to the non-treated group all PACAP-treated groups showed an increased phagocytosis as displayed by the MFI, with the highest effect observed at 1 μM concentration (control vs. 0.1 μM PACAP: *p* < 0.0001; control vs. 1 μM PACAP: *p* < 0.0001; control vs. 10 μM PACAP: *p* < 0.0001). With the signal of FITC-fluorescent microspheres being well distinguishable by flow cytometry, we were further able to subdivide the composition of each experimental group based on the number of beads being phagocyted (Figure 3B′). In line with the MFI data, all PACAP-treated groups showed an increased number of internalized microspheres when compared to the non-treated control. Moreover, treatment with 1 μM PACAP resulted in the largest fraction of BMDMs with more than 3 beads engulfed when compared to other groups (control: 68.3 ± 1.007 %; 0.1 μM PACAP: 79.33 ± 0.9939; 1 μM PACAP: 88.9 ± 1.0044 %; 10 μM PACAP: 81.3 ± 0 %). These findings were further supported by analysis of BMDMs with respect to their expression of the macrophage marker F4/80 (Figure 3C). Also here, PACAP treatment resulted in upregulation of F4/80 that was most prominent in the group treated with 1 μM PACAP (control vs. 0.1 μM PACAP: *p* < 0.0045; control vs. 1 μM PACAP: *p* < 0.0004; control vs. 10 μM PACAP: *p* < 0.004). Altogether, these results demonstrate that the neuropeptide PACAP was able to reduce the presence of *T. gondii* in peritoneal myeloid cells not by a direct anti-parasitic effect but by modulating their phagocytic capabilities.

### Decreased Parasite Burden and Expression of Inflammatory Mediators

**Figure 4:**
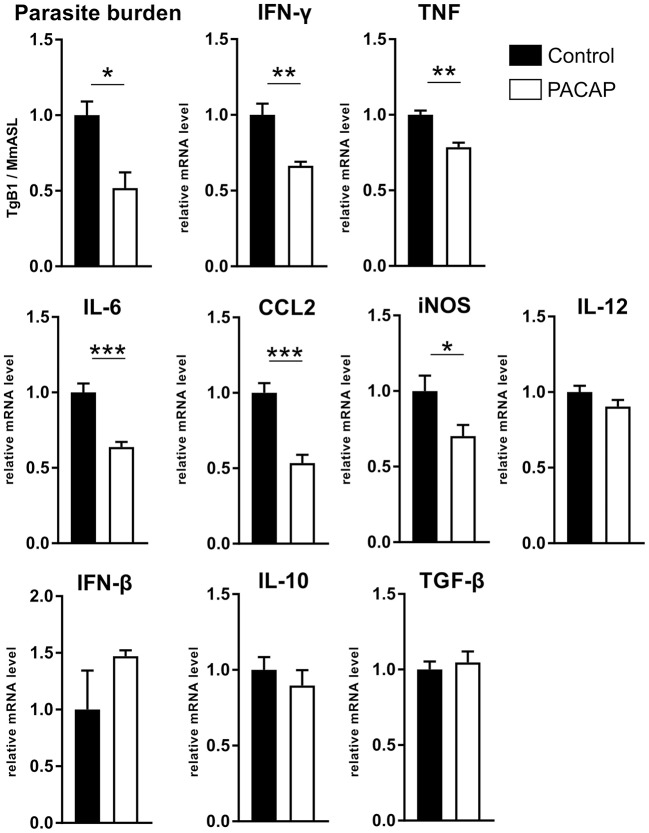
PACAP alters parasite burden and inflammatory mediators. DNA and RNA were isolated from spleens of acutely infected mice and analyzed by qPCR and RT-qPCR. The parasite burden was determined based on the presence of B1 gene of *T. gondii* (*TgB1*) normalized to the murine gene *Asl*. The gene expression levels of inflammatory mediators were determined by RT-qPCR using *Hprt* as reference gene. Data were further normalized to mean values of control group; Control (black bars) and PACAP-treated (white bars). Bar charts present results as mean ± SEM, *n* = 4. *p < 0.05, **p < 0.01, ***p < 0.001 (two-tailed unpaired *t*-test).

Previous studies have shown that PACAP inhibits the production of pro-inflammatory cytokines (Martinez et al., 1998a; Delgado et al., 1999a,b). In order to evaluate how PACAP affects the acute inflammation caused by *T. gondii*, we assessed the parasite burden and the gene expression of inflammatory mediators (Figure 4). The results show a reduced parasite load (*p* = 0.0171) in the PACAP-treated group and reduced expression of IFN-γ (*p* = 0.0022), TNF (*p* = 0.0069), IL-6 (*p* = 0.0009), CCL-2 (*p* = 0.0009), and iNOS (*p* = 0.0451). Expression levels of IL-12, IFN-β, IL-10, and TGF-β did not differ between PACAP and the control group. In summary, PACAP was able to reduce parasite burden while diminishing the robust Th1 response characteristic for *T. gondii* infection without affecting anti-inflammatory mediators.

### Immune Cells Upregulate PACAP Receptors and Neurotrophin Expression

**Figure 5:**
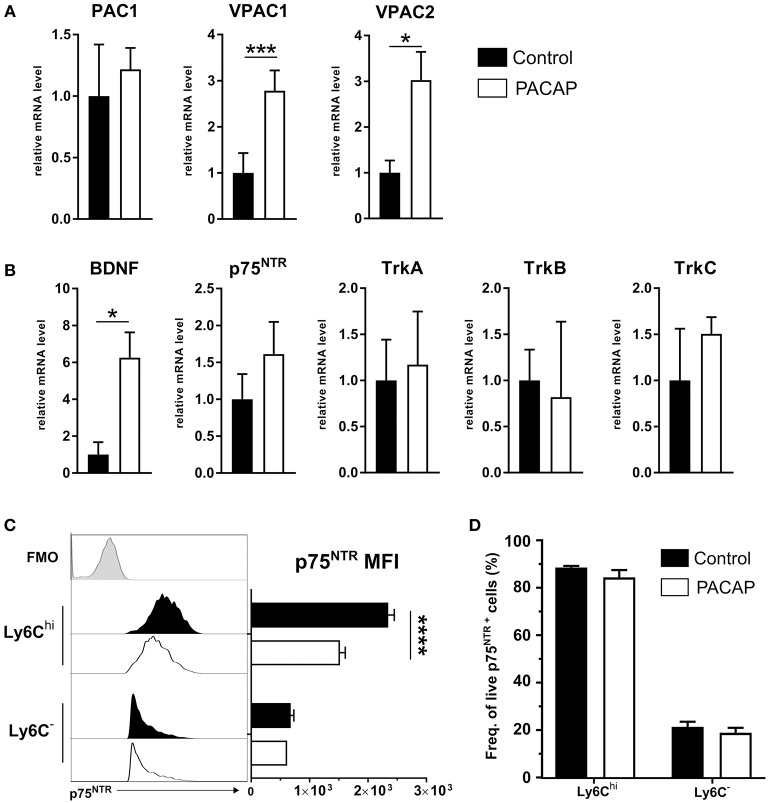
Expression of PACAP receptors, BDNF and neurotrophin receptors on peritoneal cells. Gene expression of **(A)** PACAP receptors and **(B)** BDNF and neurotrophin receptors was assessed by RT-qPCR. Control (black bars) and PACAP-treated (white bars); data are expressed as mean ± SEM, *p < 0.05, ***p < 0.001 (two-tailed unpaired *t*-test). **(C)** Flow cytometric analysis of p75^NTR^ expression on immune cell subsets; the histograms show the FMO control (light gray-tinted), control group (black-tinted) and PACAP group (black non-tinted); bar charts present the values of MFI for p75^NTR^ expression. **(D)** Frequency of cells from parent population of living, single p75^NTR+^ cells. Data are presented as mean ± SEM, *n* = 4. ****p < 0.0001 (two-tailed unpaired *t*-test).

PACAP-mediated effects on immune cells are elicited by binding of the neuropeptide to its receptors PAC1, VPAC1, and VPAC2, whereby the main anti-inflammatory effect is primarily exerted through VPAC1 resulting in activation of the cAMP/PKA pathway (Delgado et al., 2004a). This signaling pathway regulates the activity of a range of transcription factors critical for expression of the most inflammatory mediators (Delgado et al., 1998, 1999a; Delgado and Ganea, 1999, 2001). Therefore, we evaluated whether PACAP modulates the expression of its intrinsic receptors on peritoneal immune cells upon *T. gondii* infection (Figure 5A). Our results show that the neuropeptide was able to increase the expression of VPAC1 and VPAC2 ~3 fold when compared to the control group (VPAC1: *p* = 0.00001; VPAC2: *p* = 0.035). Thus, while PACAP binds to its own receptor, it also regulates their expression on innate immune cells.

Previous studies have shown the involvement of VPAC1 in myeloid cells and the PACAP-mediated signal transduction into neurotrophin signaling (El Zein et al., 2007). In general, neurotrophins are associated with the growth, development and survival of neuronal cells in the CNS (Mitre et al., 2017). However, besides neuronal tissue, neurotrophin receptors of the Trk superfamily and the p75 neurotrophin receptor (p75^NTR^) were detected in a variety of immune cells (Frossard et al., 2004; Fischer et al., 2008; Minnone et al., 2017), thus emphasizing the interdependency of the neuronal and immune system. In accordance with that, our previous work indicates that neurotrophin signaling via the p75^NTR^ affects innate immune cell behavior upon *T. gondii*-induced neuroinflammation (Dusedau et al., 2019). Therefore, to further explore the influence of PACAP-mediated signaling on the neurotrophin signaling pathway as another modulator of the innate immune response, we analyzed the expression of BDNF and neurotrophin receptors (p75^NTR^, TrkA, TrkB, and TrkC) in peritoneal exudate cells upon acute infection (Figure 5B). Surprisingly, BDNF expression level was found to be elevated ~6 fold in PACAP-treated animals when compared to the control group (BDNF, *p* = 0.0129; p75^NTR^, *p* = 0.0584; TrkA, *p* = 0.9990; TrkB, *p* = 0.9992; TrkC, *p* = 0.9674). Altogether, our results indicate an upregulation of BDNF expression upon PACAP treatment, suggesting a modulation of neurotrophin pathways in innate immune cells upon acute *T. gondii* infection.

### PACAP Reduce p75^NTR^ Expression on Ly6C^hi^ Monocytes

We have recently highlighted the upregulation of p75^NTR^ on innate immune cells in the blood and brain of infected animals in *T. gondii*-induced neuroinflammation (Dusedau et al., 2019). In the periphery, the infection increased p75^NTR^ expression on myeloid cell subsets, which are essential for control of toxoplasmosis (Dunay et al., 2010; Biswas et al., 2015). To this end, we analyzed expression of p75^NTR^ on Ly6C^hi^ inflammatory monocytes and Ly6C^−^ resident macrophages isolated from the peritoneal cavity upon acute infection. In the PACAP-treated group, the p75^NTR^ expression on Ly6C^hi^ monocytes was significantly reduced (Ly6C^hi^, *p* < 0.00001; Ly6C^−^, *p* = 0.29) (Figure 5C) with no changes in frequency of p75^NTR+^ cells for Ly6C^hi^ (*p* = 0.276) or Ly6C^−^ (*p* = 0.5181) (Figure 5D). Taken together, our results point toward the anti-inflammatory effect of PACAP on Ly6C^hi^ monocytes, and suggest the involvement of p75^NTR^ in the acute inflammatory response against *T. gondii*.

## Discussion

We hypothesized that application of the neuropeptide PACAP might modulate the behavior of myeloid-derived mononuclear cells and potentially contribute to the resolution of the infection and parasite elimination. Accordingly, we investigated the immunomodulatory effect of PACAP on innate immune cells isolated from the peritoneal cavity during experimental acute *T. gondii* infection. We detected an interaction of PACAP and neurotrophin signaling that suggests a contribution to the resolution of acute *Toxoplasmosis*.

Previously, we have reported that PACAP administration ameliorates acute small intestinal inflammation and extra-intestinal sequelae during acute ileitis caused by *T. gondii* infection (Heimesaat et al., 2014; Bereswill et al., 2019). Besides, we demonstrated the critical importance of myeloid cells to control *T. gondii* infection in the periphery as well as in the CNS (Dunay et al., 2008, 2010; Biswas et al., 2015; Mohle et al., 2016). Furthermore, our recent studies have revealed the emerging role of neurotrophin signaling via p75^NTR^ that affects innate immune cell behavior and influences structural plasticity of neurons upon *T. gondii*-induced neuroinflammation (Dusedau et al., 2019). Therefore, we analyzed the effects of PACAP on the course of acute *T. gondii* infection in mice.

Initially, our data revealed that in the peritoneal cavity, the number of recruited myeloid cells, especially Ly6C^hi^ inflammatory monocytes, was reduced by the administration of exogenous PACAP. PACAP has been shown to modulate chemokines produced by activated macrophages and adhesion molecules expressed by granulocytes, thereby affecting the recruitment of different immune cell subsets (Ganea and Delgado, 2002; El Zein et al., 2008). Under steady-state conditions, two types of macrophages are found in the peritoneal cavity: large peritoneal macrophages (LPMs) and small peritoneal macrophages (SPMs) (Ghosn et al., 2010). During inflammation, the peritoneal cell composition dramatically changes, with a massive recruitment of Ly6C^hi^ monocytes that give rise to new SPMs, while LPMs leave the peritoneal cavity and migrate to the omentum for antigen presentation (Cassado Ados et al., 2015). Upon *in vitro* LPS stimulation, SPMs developed a pro-inflammatory profile as indicated by TNF, CCL3 and RANTES production (Cain et al., 2013). These findings align with our previous results where Ly6C^hi^ monocytes contributed to *T. gondii* removal via production of TNF, iNOS and reactive oxygen species (ROS) (Dunay et al., 2008, 2010; Dunay and Sibley, 2010; Karlmark et al., 2012). Analyzing the gene expression of selected chemokines, cytokines and inflammatory mediators, we detected that exposure to PACAP diminished the levels of TNF, IL-6, and iNOS similarly to our previous observations in the intestine (Heimesaat et al., 2014). Moreover, the expression of the chemokine CCL2, important for the recruitment of Ly6C^hi^ monocytes (Biswas et al., 2015), was also significantly downregulated further supporting our observations of reduced myeloid cell recruitment. Levels of the anti-inflammatory cytokine IL-10 were not elevated, but remained balanced upon PACAP administration in our experimental setup. These data are in line with a previous finding in a study with experimental autoimmune encephalomyelitis (EAE), where PACAP was able to reduce IFN-γ levels but had no effect on production of IL-10 by spleenocytes (Kato et al., 2004).

Interestingly, we observed a decreased systemic parasite burden despite IFN-γ being negatively affected by the administration of PACAP. Generally, downregulation of IFN-γ, the main driving force against *T. gondii* infection, would result in an uncontrolled parasite replication (Suzuki et al., 1988). Contrary to previous reports with *Trypanosoma* (Delgado et al., 2009), we did not detect direct antiparasitic effects of PACAP on the *T. gondii* replication, which was assessed by monitoring changes in the size or the number of plaques. Instead, IFN-β was found to be upregulated by tendency after exposure to PACAP. The expression of the inflammatory cytokine IFN-β is upregulated following *T. gondii* infection (Mahmoud et al., 2015). Anti-parasitic effects of type I IFNs in myeloid cells are independent from iNOS and IFN-γ-induced effects. IFN-β acts via induction of IRGM1 that accumulates on the parasitophorous vacuole (PV), in order to disrupt it (Mahmoud et al., 2015). Therefore, we further analyzed the expression of IRGs (IRGM1, IRGM3, GBP2b) and other host defense factors associated with PV disruption. Here, we observed upregulated expression of IRGM1/IRGM3, which implies that PACAP modulates immune cell-mediated parasite elimination rather than direct anti-parasitic effects. Our results align with previous studies (Delgado et al., 1996a), where macrophages exhibit an increased F4/80 expression and enhanced phagocytic capacity.

In contrast to Ly6C^hi^ monocytes and Ly6G^+^ neutrophils, CD11c^+^ DC recruitment was not affected by PACAP. However, the expression of MHCII in the PACAP-treated group was increased, pointing toward a promotion of antigen recognition and subsequent activation of lymphocytes. In line with previous studies, PACAP-mediated effects on DCs have shown to be mainly induced by signaling through VPAC1, which is the same receptor-mediated pathway utilized for macrophages (Delgado et al., 2004b). Indeed, PACAP administration upregulated the expression of VPAC1 and VPAC2 but not PAC1 by peritoneal exudate cells from acutely infected mice. All three receptors result in the activation of cyclic adenosine monophosphate (cAMP) and the subsequent activation of protein kinase A (PKA) (Delgado et al., 2004a). Specific studies using agonists and antagonists for PACAP receptors have established VPAC1 as the major mediator of the immunomodulatory effects from PACAP, both *in vitro* and *in vivo*, with moderate involvement of VPAC2, and minimal or none from PAC1 (Delgado et al., 2004a). Interestingly, in human neutrophils and monocytes, PACAP interaction with VPAC1 and the NGF receptor TrkA resulted in calcium mobilization and subsequent pro-inflammatory activation (El Zein et al., 2006, 2007). Even though PACAP exposure had no effect on the gene expression of Trk receptors, we detected an upregulation of BDNF expression by immune cells upon PACAP administration.

It was previously reported that PACAP upregulated BDNF expression in primary neuronal cultures from rat cerebral cortex, as well as in human neuroblastoma cells upon injury (Frechilla et al., 2001; Shintani et al., 2005; Brown et al., 2013). BDNF is able to influence the immune system via modulation of cytokine expression in peripheral blood mononuclear cells (Vega et al., 2003). BDNF was shown to also be produced by immune cells (Kruse et al., 2007), to modulate monocyte chemotaxis, participate in tissue-healing mechanisms (Samah et al., 2008), and enhance macrophage phagocytic activity (Hashimoto et al., 2005). Recently, we have described a specific role of neurotrophins and their receptors during neuroinflammation. We described that the BDNF receptor p75^NTR^ has a functional impact on the activation status of innate immune cells during *T. gondii*-induced neuroinflammation (Dusedau et al., 2019).

Here, PACAP was able to reduce the overall recruitment of myeloid-derived mononuclear cells to the peritoneal cavity; in particular, Ly6C^hi^ monocytes. Interestingly, the same cell subset presented higher p75^NTR^ expression than Ly6C^−^ peritoneal macrophages, and the administration of PACAP exclusively reduced p75^NTR^ expression on Ly6C^hi^ monocytes. In previous studies (Lee et al., 2016), the use of an antagonist blocker of p75^NTR^ reduced the recruitment of inflammatory monocytes to the CNS, further suggesting that neurotrophin signaling is involved in immune cell migration. Moreover, in a model of in EAE model, the induction of the inflammation resulted in expression of p75^NTR^ in endothelial cells. Although they focus on endothelial p75^NTR^ expression, the study reports a differential immune cell recruitment to the CNS of p75^NTR^ knockout mice, with reduced numbers of cells from the monocyte-macrophage lineage (Kust et al., 2006). In other studies, p75^NTR^ expression by immune cells was reported to increase by a factor of 10 in response to injury (Ralainirina et al., 2010). Our previous work also showed p75^NTR^ upregulation on resident microglia cells and myeloid-derived mononuclear cell subsets in *T. gondii*-infected brains (Dusedau et al., 2019). These studies supported our findings, where immune cell recruitment and the expression of p75^NTR^ upon inflammation were reduced upon PACAP treatment.

Alongside BDNF, p75^NTR^ signaling in immune cells can be modulated by levels of neurotrophin precursors (pro-neurotrophins). proBDNF has been shown to negatively affect neuronal plasticity and cell death, reinforced by elevated levels of proBDNF detected in peripheral macrophages (Wong et al., 2010; Luo et al., 2016; Dusedau et al., 2019). Here we observed a downregulation of p75^NTR^, implying a reduced influence of pro-neurotrophins on Ly6C^hi^ monocytes and thus having a beneficial effect on the resolution of inflammation. The increase of BDNF gene expression in response to PACAP treatment and the involvement of neurotrophins/receptors with VPAC1, especially on neutrophils and monocytes, suggest a possible interaction between p75^NTR^, BDNF and PACAP within myeloid cells during an inflammatory response. However, future experiments should investigate whether the downregulation of p75^NTR^ is directly PACAP-mediated or a result of an overall reduced inflammation.

In our experiments, the effect of PACAP on parasite elimination may correlate with the synergic, indirect effect of elevated BDNF expression by peritoneal immune cells. However, the role of p75^NTR^ signaling in relation to the immune system remains poorly understood due to the complex interplay of mature vs. pro-neurotrophins, and heterodimeric interactions with Trk receptors (Meeker and Williams, 2014). In summary, our results indicate different routes of PACAP-mediated regulation of the innate response during acute *T. gondii* infection. As a potent immunomodulator, PACAP has been shown to contribute to the resolution of acute inflammation and parasite elimination by innate immune cells. Furthermore, our findings point toward a potential connection between PACAP and neurotrophin-mediated signaling in Ly6C^hi^ inflammatory monocytes. Taken together, these results contribute to the understanding of the interaction between the nervous and immune systems through neuropeptides.

## Author Contributions

CF and HD performed experiments and analyzed data. JS, NG, MD, GT, DR, and MH critically discussed experimental design, provided material, and co-edited the manuscript. ID conceived experimental design. CF and ID wrote the manuscript.

### Conflict of Interest Statement

The authors declare that the research was conducted in the absence of any commercial or financial relationships that could be construed as a potential conflict of interest.
