# Supplementary material for: Immunomodulatory Effects of the Neuropeptide Pituitary Adenylate Cyclase-Activating Polypeptide in Acute Toxoplasmosis
Source: Front Cell Infect Microbiol. 2019 May 28;9:154. doi: 10.3389/fcimb.2019.00154 (PMC6546896; doi:10.3389/fcimb.2019.00154)
Supplement: Supplementary file 1 [file Table_1.DOCX]

**Supplementary table 1. Oligonucleotide primers used for *q*PCR and RT-*q*PCR**

| **SYBR Green** | | | | |
| --- | --- | --- | --- | --- |
|  | **GenBank Accession Number** | | **Primer Sequences** | |
|  |  |  | **Sense** | **Antisense** |
| **Target genes** | | |  |  |
| *Hprt* | NM_013556 | | 5′- GCTATAAATTCTTTGCTGACCTGCTG -3′ | 5′- AATTACTTTTATGTCCCCTGTTGACTGG-3′ |
| *Adcyap1r1* (PAC1) | XM_011241153 | | 5’-GGCTGTGCTGAGGCTCTACTTTG-3’ | 5’- AGGATGATGATGATGCCGATGA -3’ |
| *Vipr1* (VPAC1) | XM_006512068 | | 5’- GATGTGGGACAACCTCACCTG -3’ | 5’- TAGCCGTGAATGGGGGAAAAC -3’ |
| *Vipr2* (VPAC2) | XM_006515806 | | 5’-GCGGTGTCTGGGACAACATC-3’ | 5’- CTGTGACATTTTCCCCAACGT -3’ |
| ***TaqMan*® Gene Expression Assay** | | | | |
| **Target gene (protein)** | | **Gene Expression Assays** | | |
| *Bdnf* (BDNF) | | Mm04230607_s1 | | |
| *Ccl2* (MCP-1) | | Mm00441242_m1 | | |
| *Gbp2b* (GPB-1) | | Mm00657086_m1 | | |
| *Hprt* (HPRT) | | Mm01545399_m1 | | |
| *Ifnb1* (IFNβ) | | Mm00439552_s1 | | |
| *Ifng* (IFN-γ) | | Mm00801778_m1 | | |
| *Igtp* (IRGM3) | | Mm00497611_m1 | | |
| *Il6* (IL-6) | | Mm00446190_m1 | | |
| *Il12a* (IL-12) | | Mm00434165_m1 | | |
| *Irgm1* (IRGM1) | | Mm00492596_m1 | | |
| *Ngfr* (p75NTR) | | Mm01309638_m1 | | |
| *Nos2* (iNOS) | | Mm00440485_m1 | | |
| *Ntrk1*(TrkA) | | Mm01219406_m1 | | |
| *Ntrk2* (TrkB) | | Mm00435422_m1 | | |
| *Ntrk3* (TrkC) | | Mm00456222_m1 | | |
| *Tnf* (TNF) | | Mm00443258_m1 | | |
|  | |  | | |
|  | |  | | |
